# The Role of Extracellular Matrix and Inflammation in the Stratification of Bleeding and Thrombotic Risk of Atrial Fibrillation on Oral Anticoagulant Therapy: Insights from Strat-Af Study

**DOI:** 10.3390/jcm12216866

**Published:** 2023-10-30

**Authors:** Francesco Alfano, Francesca Cesari, Anna Maria Gori, Martina Berteotti, Emilia Salvadori, Betti Giusti, Alessia Bertelli, Ada Kura, Carmen Barbato, Benedetta Formelli, Francesca Pescini, Enrico Fainardi, Stefano Chiti, Chiara Marzi, Stefano Diciotti, Rossella Marcucci, Anna Poggesi

**Affiliations:** 1 Department of Experimental and Clinical Medicine, University of Florence, 50134 Florence, Italy; francescoalfa12@gmail.com (F.A.); francesca.cesari@gmail.com (F.C.); annamaria.gori@unifi.it (A.M.G.); martina.berteotti@gmail.com (M.B.); betti.giusti@unifi.it (B.G.); alessia.bertelli@unifi.it (A.B.); ada-kura@hotmail.it (A.K.); 2 Center for Atherothrombotic Diseases, Careggi University Hospital, 50134 Florence, Italy; 3 NEUROFARBA Department, Neuroscience Section, University of Florence, 50134 Florence, Italy; emilia.salvadori@unifi.it (E.S.); carmenbarbato88@gmail.com (C.B.); benedetta.formelli@unifi.it (B.F.); anna.poggesi@unifi.it (A.P.); 4 Stroke Unit, Careggi University Hospital, 50134 Florence, Italy; francesca.pescini@unifi.it; 5 Neuroradiology Unit, Careggi University Hospital, Department of Experimental and Clinical Biomedical Sciences, University of Florence, 50134 Florence, Italy; henryfai@tin.it; 6 Health Physics Unit, Careggi University Hospital, 50134 Florence, Italy; stefano.chiti@gmail.com; 7 Institute of Applied Physics “Nello Carrara” (IFAC), National Research Council of Italy (CNR), 50019 Sesto Fiorentino, Italy; chiara.marzi3@unibo.it; 8 Department of Electrical, Electronic, and Information Engineering “Guglielmo Marconi”, University of Bologna, 40126 Bologna, Italy; stefano.diciotti@unibo.it

**Keywords:** atrial fibrillation, biomarkers, extracellular matrix, cerebral infarcts, hyperintensities of white matter, SVDs, cerebral microbleeds, EPVS

## Abstract

In anticoagulated atrial fibrillation (AF) patients, the validity of models recommended for the stratification of the risk ratio between benefits and hemorrhage risk is limited. We hypothesize that both circulating and neuroimaging-based markers might improve the prediction of bleeding and thrombotic risk in anticoagulated AF patients. The Strat-AF study is an observational, prospective, single-center study enrolling 170 patients with AF; recruited patients are evaluated by means of a comprehensive protocol, with clinical, cerebral magnetic resonance imaging and circulating biomarkers assessment. The main outcome is the evaluation of cerebral microangiopathy related to the levels of circulating biomarkers of inflammation and extracellular matrix (ECM) remodeling. At multivariate logistic regression analysis adjusted for age, sex, CHA2DS2-VASc, HAS-BLED and type of anticoagulant, matrix metalloproteinases (MMP)-2 levels were significantly and positively associated with the presence of cerebral microbleeds (CMBs). A significant association between MMP-2, tissue inhibitor of metalloproteinases (TIMP)-1,-2,-4 levels and white matter hyperintensity was also found. Concerning the small vessel disease (SVD) score, MMP-2 and TIMP-1,-2 levels were associated with the presence of two and three or more signs of SVD, whereas TIMP-4 levels were associated with the presence of three signs of SVD with respect to patients with no instrumental signs of SVD. As regarding the presence of enlarged perivascular spaces (EPVS), a significant association was found for high levels of interleukin (IL)-8 and TIMP 1-2-3. These results demonstrate that patients with AF have evidence of impaired ECM degradation, which is an independent risk factor for thrombotic complications of AF patients on oral anticoagulant therapy. The incorporation of these markers in the prognostic schemes might improve their clinical capability in predicting stroke risk and thrombotic complications.

## 1. Introduction

Atrial fibrillation (AF) is the most common supraventricular arrhythmia encountered in clinical practice and is associated not only with increased risk of stroke and systemic embolism, but also with increased morbidity and mortality [1]. The currently estimated prevalence of AF in adults is between 2% and 4% [2] and a 2.3-fold rise [3] is expected [4], owing to extended longevity in the general population [5]. Anticoagulant therapies reduce thromboembolic risk in AF, despite increasing bleeding risk. At present, thrombotic and bleeding risk stratification schemes are aimed at identifying patients who may benefit from oral anticoagulation. However, the validity of such schemes relies only on clinical information, which needs an improvement, in particular in the capacity of identifying the bleeding risk. Emerging evidence suggests a significant role of inflammation in the pathogenesis of AF [6]. In addition, several studies have described an association between AF and abnormal prothrombotic plasma markers, including fibrinogen, von Willebrand factor (vWF), and soluble P-selectin, suggesting that the arrhythmia itself contributes to the development of a pro-thrombotic state [7]. Furthermore, various inflammatory markers and mediators such as C-reactive protein (CRP), tumor necrosis factor alpha (TNF)-alpha, interleukin (IL)-2, IL-6 and IL-8 have been linked with both the presence and the outcomes of AF [8].

Clinical and experimental studies have reported a correlation between AF, atrial fibrosis [9] and cardiac remodeling, demonstrating an impaired matrix degradation in AF patients [10]. In the extracellular matrix degradation, the balance between matrix metalloproteinases (MMPs) and tissue inhibitors of metalloproteinases (TIMPs) is crucial and previous studies reported a relationship between the MMP/TIMP system and prothrombotic state. 

In addition, advanced imaging technology such as magnetic resonance imaging (MRI) has led to the increased detection of asymptomatic brain changes in AF patients [11,12], mainly those related to small vessel disease (SVD) and cerebral microbleeds [13].

On this basis, the contemporary evaluation of circulating biomarkers and MRI lesions could offer new evidence in the prediction of both thrombotic and hemorrhagic risk in AF patients, in order to improve the management of therapy and the reduction of long-term complications that a non-optimal control of the disease brings with it. 

The aim of our study was to evaluate, in a population of AF patients undergoing oral anticoagulant therapy, the association between circulating biomarkers involved in the pathogenesis of AF and the presence of brain MRI lesions related to SVD (non-lacunar infarcts, lacunar infarcts, presence of cerebral microbleeds, presence of enlarged perivascular spaces and hyperintensities of white matter) [14].

## 2. Materials and Methods

### 2.1. Study Population

The Strat-AF study (Stratification of cerebral bleeding risk in AF) is an observational, prospective, single-center, hospital-based study enrolling elderly patients with AF, referred from the outpatient-clinic of Atherothrombotic Disease Center of Careggi University Hospital, where they are followed for the management of oral anticoagulation therapy in primary or secondary prevention of thromboembolic events. The main aim is to evaluate the added value of both circulating and brain MRI biomarkers on the prediction of cerebral bleeding and thrombotic risk in AF patients under treatment with oral anticoagulants (OACs). For this purpose, patients ≥65 years with a diagnosis of AF, ongoing OAC with vitamin K antagonists (VKA) or direct OACs (DOACs) and with no contraindications to undergo MRI were enrolled.

The study was conducted in accordance with the Declaration of Helsinki and ethical approval was obtained by the Ethics Committee of Careggi University Hospital (project identification code 16RFAP, approved on March 2017); all participants gave written informed consent for inclusion before enrollment. The study design and methodology have been previously described [14]. 

For the purpose of this paper, demographic characteristics (age, sex, years of education), previous stroke events, vascular risk factors and comorbidities (hypertension, diabetes, dyslipidemia, physical activity, smoking habits, alcohol consumption, peripheral arterial disease, ischemic heart disease, myocardial infarction, heart failure) were collected and used for the analysis.

The CHA2DS2-VASc and HAS-BLED scores were calculated based on the clinical information. The CHA2DS2-VASc scores range from 0 to 9 and are based on the following clinical information: congestive heart failure (score 1), hypertension or antihypertensive therapy (score 1), age ≥ 75 years (score 2), diabetes (score 1), stroke (score 2), vascular disease (score 1), age 65–74 years (score 1), and sex category (female, score 1) [15]. The HAS-BLED scale was used to estimate the bleeding risk, with scores ranging from 0 to 9, which was calculated based on the following clinical information: uncontrolled hypertension (systolic blood pressure >160 mm Hg, score 1), abnormal renal and/or hepatic function (score 1 or 2), stroke (score 1), bleeding history or predisposition (score 1), labile international normalized ratio (score 1), age (score 1), and drugs or excessive alcohol drinking (score 1 or 2) [16].

The study protocol was approved by each ethical committee, and all patients gave informed consent.

### 2.2. Laboratory Determinations

Whole venous blood was collected in tubes without anticoagulant and with citrated whole blood (3.2%, 0.109 M). Tubes without and with anticoagulant were centrifuged at room temperature at 1500 g for 15 min, and the supernatants were stored in aliquots at −80 °C until the measurement of blood biomarkers, which was performed six months after the enrollment.

Samples were analyzed in a unique central laboratory. Levels of different inflammatory markers [interleukin (IL)-4, IL-6, IL-8, IL-10, Tumor Necrosis Factor alpha (TNF-alpha), chemokine (C-C motif) ligand 2 (CCL2) also referred to as monocyte chemoattractant protein 1, C-X-C motif chemokine ligand 10 (CXCL10) also known as Interferon gamma-induced protein 10, Intercellular Adhesion Molecule-1 (ICAM-1), Vascular cell adhesion protein 1 (VCAM-1) and Vascular-Endothelial Growth Factor (VEGF)] were determined on serum samples using a Bio-Plex suspension array system and R&D Kits (R&D System, Milan, Italy). Metalloproteinases (MMP-2, MMP-7, MMP-8, MMP-9, MMP-12), extracellular matrix metalloproteinase inducer (EMMPRIN) and tissue inhibitors of metalloproteinases (TIMP-1, TIMP-2, TIMP-3 and TIMP-4) were assessed in the same serum samples using a Bio-Plex suspension array system (Bio-Rad Laboratories Inc., Hercules, CA, USA) and R&D Kits (R&D System, Milan, Italy) according to the manufacturer’s instructions. The coefficients of variation of inflammatory markers, MMPs and TIMPs assays were <6%.

As regards clotting parameters, the activity of VWF was determined on citrated plasmas by a latex particle-enhanced immunoturbidimetric assay (Werfen, Milan, Italy). PAI-1 Antigen levels were assessed on plasma samples by immunoenzymatic assay (Hyphen Biomed, Neuville-sur-Oise, France).

### 2.3. Neuroimaging Assessment

Brain MRI have been performed on a 1.5 T MRI (Ingenia, Philips Healthcare, Best, The Netherlands). The MRI protocol included the following sequences: sagittal T1-weighted spin-echo [repetition time (TR) = 547 ms; echo time (TE) = 12 ms; slice thickness = 5 mm; interslice spacing = 0.5 mm; matrix size = 320 × 250; field of view (FOV) = 23 cm × 23 cm; number of signals averaged (NSA) = 1], coronal T2-weighted fast spin-echo (TR = 3347 ms; TE = 110 ms; slice thickness = 5 mm; interslice spacing = 0.5 mm; matrix size = 512 × 322; FOV = 22 cm × 22 cm; NSA = 2); axial fluid-attenuated inversion recovery (FLAIR) [TR = 11,000 ms; TE = 125 ms; inversion time (TI) = 2800 ms; slice thickness = 5 mm; interslice spacing = 0.5 mm; matrix size = 384 × 204; FOV = 23 cm × 23 cm; NSA = 2]; axial gradient-echo T2∗ (GRE) [TR = 534 ms; TE = 23 ms; flip angle (FA) = 18; slice thickness = 5 mm; interslice spacing = 0.5 mm; matrix size = 256 × 185; FOV = 23 cm × 23 cm; NSA = 1]; axial diffusion-weighted imaging (DWI) (TR = 3891 ms; TE = 75 ms; slice thickness = 5 mm; interslice spacing = 0.5 mm; matrix size = 164 × 162; FOV = 23 cm × 23 cm; NSA = 2); gradient-echo 3D T1-weighted (TR = 7.5 ms; TE = 3.4 ms; TI = 950, slice thickness = 1 mm; matrix size = 256 × 241; FOV = 25.6 cm × 25.6 cm; NSA = 1) followed by multiplanar reconstruction (MPR) in axial, coronal, and sagittal planes.

MRI was performed within 3 weeks from the blood sample collection [median and interquartile range: 13 (7–21) days].

The brain imaging protocol was planned and set up by imaging personnel with different expertise and skills, as suggested by current guidelines [17].

Cerebral lesion burden was visually assessed by two trained and experienced raters using validated scales. In particular, each MRI scan was evaluated by an expert neuroradiologist and a stroke neurologist. Both the neuroradiologist and the neurologist were blinded to any clinical information of the patients and/or results of biomarkers investigated.

Cerebrovascular lesion burden encompassed markers of small vessel disease (SVD) and large vessel disease and included the following markers:

- Non-lacunar infarcts: defined as cortical or subcortical (>15 mm in diameter) lesions in vascular territories, they were numerically rated on T1-weighted and T2-FLAIR sequences.
- SVD markers were selected and evaluated according to the STRIVE criteria [18], and included:
  - White matter hyperintensities (WMH), rated on axial FLAIR sequences using the modified Fazekas scale [19] which defines three different grades of deep WMH severity: mild (single lesions < 10 mm; areas of “grouped” lesions < 20 mm in any diameter), moderate (single hyperintense lesions between 10 and 20 mm; areas of “grouped” lesions ≥ 20 mm in any diameter; no more than “connecting bridges” between individual lesions), and severe (single lesions or confluent areas of hyperintensity ≥ 20 mm in any diameter).
  - Cerebral microbleeds (CMBs), rated on axial gradient-echo T2-weighted sequences according to the Microbleeds Anatomical Rating Scale (MARS) [20], which identifies “definite” microbleeds as small, rounded or circular, well-defined hypointense lesions within brain parenchyma with clear margins ranging from 2 to 10 mm in size and classified location as deep, infratentorial and lobar.
  - Lacunar infarcts: lacunes of presumed vascular origin were detected and counted on T1 and T2 FLAIR sequences; they are defined as small (3–15 mm in diameter) round or ovoid, subcortical, fluid-filled cavities, usually surrounded by a hyperintense rim.
  - Enlarged perivascular spaces (EPVS), defined as fluid-filled spaces following small vessels course, with round shape < 3 mm of diameter at basal ganglia (bgEPVS) level, hypointense in axial T1 images and rated estimating their absolute number in 3 slices of that anatomical region on the more injured side, then categorized in the five-level scale: 0 = none, 1 = 1–10, 2 = 11–20, 3 = 21–40, 4 = >40 PVS per region [21].
  - SVD score (range 0–4: 0 = no signs, 1 = 1 sign, 2 = 2 signs, 3 = 3 signs, 4 = ≥3 signs), which incorporates 4 established neuroimaging biomarkers of SVD and aims to capture the overall burden of cerebral SVD [22]. The score was calculated as follows: presence of ≥1 lacunes (+1 point); presence of ≥1 MBs (+1 point); moderate/abundant (grade 2–4) bgEPVS (+1 point); moderate to severe WMHs evaluated as confluent deep Fazekas Score 2 or 3 or periventricular Fazekas Score 3 (+1 point).

### 2.4. Statistical Analysis

As a main explanatory variable, we used the baseline of inflammatory markers, MMPs and TIMPs, vWF and PAI-1 antigen levels. Differences in these biomarker values were analyzed in relation to demographic and clinical features and across subgroups of patients with different outcomes.

We used Pearson χ2 to test for significance while comparing binary variables and ANOVA or Kruskal–Wallis H Test for numeric variables as appropriate. Values are presented as median and interquartile range if they had a non-Gaussian distribution.

To analyze differences in biomarker levels, we chose the Mann–Whitney U Test because of relatively large statistical variations. The net effect of each biomarker’s baseline on outcomes was then estimated by a logistic regression model including as covariates age, sex, CHA2DS2-VASc, HAS-BLED and type of anticoagulant.

We chose variables for the adjustment of multivariate logistic regression analysis according to the significance at univariate analysis. We added in the multivariate analyses model the variables CHA2DS2-VASc and HASBLED as these variables are associated with SVD lesions (microbleeds, lacunar and non-lacunar infarcts and SVD score). In addition, we adjusted also for the type of anticoagulants as our population is not homogenous in terms of anticoagulation and therefore, we had to control the possible influence of the different types of anticoagulants on the circulating biomarkers.

To correct results for multiple comparisons, we used the false discovery rate testing in all the statistical analyses. 

A significant level was defined as *p* < 0.05. All analyses were performed with SPSS 20.0 (SPSS Inc., Chicago, IL, USA) and Stata 13.0 (Lakeway Dr, College Station, TX, USA).

## 3. Results

The results refer to 170 subjects (mean age 77.7 ± 6.8 years, females *n* = 59, 34.7%) enrolled in the Strat-AF Study with complete clinical, biomarker and brain MRI information. Demographic and clinical characteristics, as well as the main cardiovascular risk factors and comorbidities, are shown in Table 1. The subjects of the study were all on oral anticoagulant therapy: 30.6% (*n* = 52) were on VKA, whereas 69.4% (*n* = 118) were on DOACs. Regarding those who were on AVK, 81.3% (*n* = 39) of them had an adequate TTR (>60%), whereas, with regard to patients anticoagulated with DOACs, we performed the dosage of each type of DOAC (apixaban, dabigatran, edoxaban, rivaroxaban) and discovered that in the majority of cases (>90%), the DOAC concentrations were in line with the average concentration reported in clinical studies. Concerning the distributions of neuroimaging characteristics: a moderate-to-severe degree of WMH was present in 67.1% (*n* = 114) of the total cohort, at least one lacunar infarct in 21.7% (*n* = 37), at least one non-lacunar infarct in 34.7% (*n* = 59), at least one CMB in 17.1% (*n* = 29) and at least one sign of SVD in 83.5% (*n* = 142); EPVS were present in 58.8% (*n* = 100).

### 3.1. Circulating Biomarkers According to Cerebral Alterations by MRI Evaluation

#### 3.1.1. Lacunar and Non-Lacunar Infarcts

As shown in Table 2, patients with lacunar infarcts had significantly higher levels of IL-8 and vWF, with respect to patients without (*p* = 0.035). Similarly, patients with lacunar infarcts had higher levels of TIMP-1, TIMP-2 and TIMP-3 (*p* = 0.003, 0.031 and 0.035, respectively).

In patients with non-lacunar infarcts, we detected significantly higher PAI-1 levels than patients without non-lacunar infarcts (*p* = 0.008). 

#### 3.1.2. Cerebral Microbleeds

Patients with CMBs had higher levels of MMP-2 and of TIMP-2,-3 with respect to patients without CMBs, as shown in Table 3. IL-8 circulating levels were higher, but not significantly (*p* = 0.087), in patients with CMBs with respect to patients without CMBs. The other biomarkers did not significantly differ between patients with or without CMBs.

#### 3.1.3. White Matter Hyperintensity (WMH)

As shown in Table 4, in patients with a moderate-to-severe degree of WMH, we detected significantly higher circulating levels of IL-6 (*p* = 0.040). Similarly, the circulating levels of MMP-2, TIMP-1, TIMP-2, TIMP-3 and TIMP-4 were also significantly higher in patients with moderate/severe grade of WMH with respect to patients with an absent or mild degree of WMH (*p* < 0.001, *p* = 0.041, *p* = 0.019, *p* = 0.019 and *p* = 0.039, respectively). 

#### 3.1.4. Enlarged Perivascular Spaces Basal Ganglia (bgEPVS) 

Patients with basal ganglia EPVS had significantly higher levels of IL-8, TIMP-1, TIMP-2, and TIMP-3 (*p* = 0.021, *p* = 0.003, *p* = 0.004 and *p* = 0.048, respectively). We also found higher, even if not statistically significant, levels of IL-6, MMP-12 and TIMP-4 in patients with bgEPVS with respect to patients without bgEPVS (Table 5).

#### 3.1.5. SVD Score

According to the presence of one, two and three cerebral MRI signs of SVD, we detected higher circulating levels of IL-6, IL-8 and VCAM-1, even though the statistical significance is not reached (*p* = 0.093, *p* = 0.074 and *p* = 0.066, respectively). Circulating levels of MMP-2, MMP-12, TIMP-1, TIMP-2, TIMP-3 and TIMP-4 were significantly associated with the presence of one, two and three cerebral MRI signs of SVD (*p* < 0.001, *p* = 0.041, *p* < 0.001, *p* < 0.001, *p* = 0.019 and *p* = 0.039, respectively) (Table 6).

### 3.2. Multivariate Analyses

In order to establish an independent association between circulating biomarkers and the presence of brain MRI lesions, we performed a multivariate logistic regression analysis adjusted for age, sex, CHA2DS2-VASc, HAS-BLED and type of anticoagulant.

As shown in Table 7, there was a non-significant tendency towards the association between the higher levels of TIMP-2,-3, vWF and the presence of lacunar infarcts. 

At the multivariate analysis, PAI- 1 levels did not remain significantly associated with the presence of non-lacunar infarcts.

Concerning metalloproteinases and their inhibitors, at multivariate logistic regression analysis, only MMP-2 remained significantly and positively associated with the presence of CMB (*p* = 0.023). A non-significant tendency towards the association between the higher levels of TIMP-2, TIMP-3 and CMB was also found.

The association between high circulating levels of MMP-2 and TIMPs and MRI lesions was also evidenced by the relation with the hyperintensity of white matter. In fact, elevated MMP-2 and TIMP-1,-2,-4 levels remained significantly associated with white matter hyperintensity (*p* = 0.025, *p* = 0.005, *p* = 0.008 and *p* = 0.012, respectively), also at the multivariate logistic regression analysis, as shown in Table 7. After adjustment for confounder factors, IL-6 levels did not remain significantly associated with the WMH.

At multivariate regression analysis, the presence of bgEPVS was significantly and positively associated with circulating levels of all four TIMPs (1, 2, 3 and 4) and of IL-8 (*p* = 0.012, *p* = 0.035, *p* = 0.036, *p* = 0.015 and *p* = 0.024, respectively). 

Concerning the SVD score, elevated levels of MMP-2, TIMP-1 and TIMP-2 were associated with the presence of two and three or more signs of SVD with respect to patients with no instrumental signs of SVD. In addition, elevated levels of TIMP-4 were associated with the presence of three or more signs of SVD with respect to patients with no instrumental signs of SVD (Table 7). A non-significant tendency towards the association between the higher levels of IL-8 and the presence of two or more signs of SVD was also documented.

**Table 2:** Circulating biomarkers in relation with the presence of lacunar and non-lacunar infarcts. Results are expressed as median (range).

		Circulating Biomarkers																					
		IL-6 (pg/mL)	IL-8 (pg/mL)	TNF-α (pg/mL)	IL-4 (pg/mL)	IL-10 (pg/mL)	CCL-2 (pg/mL)	CXCL-10 (pg/mL)	ICAM-1 (ng/mL)	VCAM-1 (ng/mL)	VEGF (pg/mL)	EMMPRIN (ng/mL)	MMP-2 (ng/mL)	MMP-7 (ng/mL)	MMP-8 (ng/mL)	MMP-9 (ng/mL)	MMP-12 (ng/mL)	TIMP-1 (ng/mL)	TIMP-2 (ng/mL)	TIMP-3 (ng/mL)	TIMP-4 (ng/mL)	VWF (%)	PAI-1 (ng/mL)
**Lacunar infarcts**	**Absent** (*n* = 133)	1.56 (0.38–3.3)	**8.05** **(4.69–12.5)**	2.3 (1.06–4.14)	12.81 (5–29.94)	2.98 (0.39–3.56)	317.36 (223.36–440.37)	15.04 (10.06–23.8)	331.55 (261.14–468.64)	1470.4 (1001.75–2117.95)	60.43 (35.15–106.02)	5.42 (4.03–6.65)	511.24 (412.7–630.4)	5.48 (2.86–7.04)	7.46 (3.51–13.58)	308.72 (182.75–456.16)	403.1 (69.04–594.96)	**158.22** **(126.67–202.9)**	**124.03** **(96.56–166.3)**	**33.52** **(24.52–50.02)**	3.1 (2.27–4.2)	**153.7** **(127.9–203.7)**	9.16 (7.2–15.4)
	**Present** (*n* = 37)	1.56 (0.38–2.73)	**9.8** **(7.29–14.94)**	2.03 (0.73– 3.5)	12.81 (5.5–35.8)	2.89 (0.24–3.49)	332.38 (264.7–417.65)	14.51 (10.89–21.45)	332.93 (280.89–626.47)	1290.5 (1069.4–1894.15)	79.14 (47.33–118.47)	5.66 (3.73–7.46)	532.82 (454.17–653.76)	5.37 (3.39–6.78)	8.46 (4.21–16.67)	295.33 (182.39–565.52)	257.88 (54.66–511.11)	**184.23** **(160.47–243.29)**	**145.99** **(120–205.24)**	**46.09** **(28.57–62.22)**	3.36 (2.6–6.2)	**199.6** **(148.8–222.25)**	9.35 (6.6–15)
	***p***	0.684	**0.035**	0.449	0.97	0.093	0.605	0.904	0.534	0.543	0.11	0.594	0.292	0.697	0.514	0.67	0.365	**0.003**	**0.031**	**0.035**	0.151	**0.024**	0.604
**Non-lacunar infarcts**	**Absent** (*n* = 111)	1.73 (0.38–3.25)	8.56 (4.72–13.15)	2.21 (0.73–4)	12.81 (5–29.4)	3 (0.3–3.56)	328.41 (230.45–445.02)	13.73 (9.8–21.15)	332.97 (264.18–463.87)	1424.4 (1003.5–2044.3)	66.41 (35.83–106.75)	5.51 (4.03–6.87)	509.52 (412.15–629.03)	5.22 (2.99–6.58)	7.98 (3.85–15.94)	315.89 (189.93–502.98)	391.61 (65.32–594.96)	162.8 (128.48–205)	124.79 (96.29–168.47)	35.51 (23.73–53.04)	3.16 (2.24–4.58)	150.75 (126.4–204.58)	**8.62** **(6.78–12.03)**
	**Present** (*n* = 59)	1.56 (0.3– 2.73)	8.18 (5.77–12.23)	2.04 (1.06–4.14)	12.81 (6–35.81)	2.89 (0.44–3.51)	317.36 (228.93–400.7)	16.31 (11.26–24.14)	326.67 (263.97–554.96)	1350 (1011.9–2094.6)	60.43 (43.45–112.62)	5.42 (3.86–6.98)	520.07 (450.5–655.8)	5.72 (3.1–7.14)	7.33 (3.18–11.77)	271.99 (173.21–389.66)	330.34 (46.35–594.96)	165.53 (135.49–232.17)	138.15 (108.92–192.16)	36.94 (27.56–61.7)	2.95 (2.48–4.82)	193.4 (140.65–218.43)	**12.02** **(7.72–17.67)**
	***p***	0.617	0.956	0.702	0.322	0.877	0.546	0.173	0.865	0.74	0.488	0.820	0.36	0.277	0.224	0.133	0.474	0.292	0.172	0.345	0.678	0.488	**0.008**

**Table 3:** Circulating biomarkers in relation with the presence of cerebral microbleeds (CMBs). Results are expressed as median (range).

		Circulating Biomarkers																					
		IL-6 (pg/mL)	IL-8 (pg/mL)	TNF-α (pg/mL)	IL-4 (pg/mL)	IL-10 (pg/mL)	CCL-2 (pg/mL)	CXCL-10 (pg/mL)	ICAM-1 (ng/mL)	VCAM-1 (ng/mL)	VEGF (pg/mL)	EMMPRIN (ng/mL)	MMP-2 (ng/mL)	MMP-7 (ng/mL)	MMP-8 (ng/mL)	MMP-9 (ng/mL)	MMP-12 (ng/mL)	TIMP-1 (ng/mL)	TIMP-2 (ng/mL)	TIMP-3 (ng/mL)	TIMP-4 (ng/mL)	VWF (%)	PAI-1 (ng/mL)
**CMBs**	**Absent** (*n* = 141)	1.56 (0.38–3.32)	8.18 (4.69–12.08)	2.04 (1.06–4)	12.81 (5–30.48)	2.89 (0.32–3.56)	322.38 (232.3–443.7)	14.76 (10.54–22.65)	332.19 (265.73–486.03)	1406.2 (1005–2035.3)	63.03 (37.11–110.97)	5.41 (4.08–6.56)	**500.59** **(411.9–610.88)**	5.36 (2.99–6.7)	7.69 (3.68–13.78)	307.43 (182.75–463.62)	319.73 (62.96–586.8)	161.38 (131.83–199.5)	**124.79** **(96.71–163.77)**	**34.09** **(23.35–50.64)**	2.94 (2.30–4.15)	173.6 (129.25–209.05)	9.01 (6.97–16.56)
	**Present** (*n* = 29)	1.73 (0.34–2.73)	9.49 (6.69–15.29)	2.53 (0.63–4.75)	6.5 (5.25–33.37)	3 (0.29–3.65)	332.38 (226.76–382.26)	14.91 (10.29–22.96)	314.15 (248.81–551.82)	1500 (1034.48–2121.15)	75.34 (41.9–111.89)	6.24 (3.54–7.64)	**621.75** **(501.03–781.09)**	5.72 (3.1–7.9)	7.31 (3.4–14.77)	295.33 (178.06–464.2)	414.58 (72.44–612.28)	202.94 (133.24–238.6)	**157.44** **(121.17–242.82)**	**41.46** **(30.24–65.81)**	3.82 (2.56–6.26)	159.8 (129.7–212.5)	9.59 (7.05–12.94)
	***p***	0.868	0.087	0.954	0.868	0.927	0.411	0.921	0.695	0.817	0.600	0.329	**0.002**	0.544	0.83	0.786	0.338	0.062	**0.011**	**0.027**	0.1	0.898	0.82

**Table 4:** Circulating biomarkers in relation with the presence of white matter hyperintensities (dichotomous Fazekas). Results are expressed as median (range).

		Circulating Biomarkers																					
		IL-6 (pg/mL)	IL-8 (pg/mL)	TNF-α (pg/mL)	IL-4 (pg/mL)	IL-10 (pg/mL)	CCL-2 (pg/mL)	CXCL-10 (pg/mL)	ICAM-1 (ng/mL)	VCAM-1 (ng/mL)	VEGF (pg/mL)	EMMPRIN (ng/mL)	MMP-2 (ng/mL)	MMP-7 (ng/mL)	MMP-8 (ng/mL)	MMP-9 (ng/mL)	MMP-12 (ng/mL)	TIMP-1 (ng/mL)	TIMP-2 (ng/mL)	TIMP-3 (ng/mL)	TIMP-4 (ng/mL)	VWF (%)	PAI-1 (ng/mL)
**White matter hyperintensities**	**Absent -Mild** (*n* = 56)	**1.19** **(0.3–2.87)**	8.54 (5.65–13.56)	2.03 (0.69–3.43)	6.5 (4.78–24.9)	2.97 (1.19–3.56)	329.01 (215.92–452.54)	14.15 (9.42–23.51)	317.89 (270.37–410.29)	1321.35 (974.18–1951.5)	66.92 (33.40–111.9)	5.67 (4.35–6.87)	**456.23** **(383.95–553.42)**	5.53 (2.88–7.17)	7.2 (2.45–12.8)	269.29 (167.16–408.5)	257.88 (62.96–463.5)	**148.28** **(11.38–187.2)**	**120.91** **(96.1–152.18)**	**30.49** **(21.36–45.3)**	**2.66** **(2.28–3.63)**	174.6 (128–207.13)	8.99 (6.88–15.1)
	**Moderate- Severe** (*n* = 114)	**1.79** **(0.58–3.5)**	8.07 (4.98–12.8)	2.53 (1.06–4.3)	12.81 (5.58–35.8)	2.89 (0.31–3.56)	321.57 (232.29–424.2)	15.79 (10.9–21.89)	337.99 (255.38–552.8)	1479.5 (1050.45–2095.5)	61.07 (38.88–109.23)	5.42 (3.85–6.88)	**546.97** **(452.48–685.97)**	5.4 (3.1–6.77)	7.88 (4.19–14.53)	321,58 (201.35–468.88)	414.58 (62.96–594.96)	**173.13** **(140.96–220.9)**	**136.96** **(105.5–198.28)**	**38.7** **(27.56–58.79)**	**3.37** **(2.38–5.46)**	161.85 (130.13–212.48)	9.73 (7.1–16.49)
	***p***	**0.040**	0.584	0.252	0.273	0.605	0.923	0.315	0.624	0.162	0.604	0.683	**<0.001**	0.884	0.437	0.181	0.128	**0.041**	**0.019**	**0.019**	**0.035**	0.760	0.746

**Table 5:** Circulating biomarkers in relation with the presence of enlarged perivascular spaces (EPVS). Results are expressed as median (range).

		Circulating Biomarkers																					
		IL-6 (pg/mL)	IL-8 (pg/mL)	TNF-α (pg/mL)	IL-4 (pg/mL)	IL-10 (pg/mL)	CCL-2 (pg/mL)	CXCL-10 (pg/mL)	ICAM-1 (ng/mL)	VCAM-1 (ng/mL)	VEGF (pg/mL)	EMMPRIN (ng/mL)	MMP-2 (ng/mL)	MMP-7 (ng/mL)	MMP-8 (ng/mL)	MMP-9 (ng/mL)	MMP-12 (ng/mL)	TIMP-1 (ng/mL)	TIMP-2 (ng/mL)	TIMP-3 (ng/mL)	TIMP-4 (ng/mL)	VWF (%)	PAI-1 (ng/mL)
**EPVS**	**Absent** (*n* = 70)	1.21 (0.3–2.93)	**6.89** **(4.56–10.65)**	2.03 (1.06–3.63)	6.70 (4.78–24.9)	2.89 (0.3–3.56)	299.8 (216.7–391.25)	16.07 (10.86–23.15)	329.11 (270.47–450.79)	1347.80 (1007.2–1910)	61.21 (36.88–94.33)	5.40 (3.85–6.58)	489.31 (425.28–620.19)	5.93 (3.73–7.27)	7.26 (3.50–13.85)	283.98 (174.67–435.03)	257.88 (46.35–471.47)	**149.21** **(115.35–194.56)**	**117.78** **(91.55–148.06)**	**32.05** **(17.66–52.71)**	2.86 (2.17–4.00)	154.3 (129.6–203.70)	9.36 (6.89–21.23)
	**Present** (*n* = 100)	1.73 (0.78–3.96)	**8.04** **(5.94–14.32)**	2.37 (10.81–4.22)	12.81 (5.58–35.8)	2.95 (0.3–3.49)	334.77 (233.09–458.7)	14.43 (10.38–22.62)	330.41 (256.14–542.46)	1486.75 (1000.88–2180.4)	63.99 (37.52–112.26)	5.67 (4.35–7.58)	536.59 (430.19–635.19)	5.02 (3.03–6.78)	7.90 (3.99–13.66)	319.50 (191.56–463.76)	450.10 (81.91–594.96)	**172.00** **(148.23–212.03)**	**139.07** **(111.99–192.16)**	**38.82** **(27.56–55.99)**	3.18 (2.40–5.45)	170.45 (127.08–212.1)	8.75 (7.1–15.78)
	***p***	0.052	**0.021**	0.516	0.490	0.939	0.124	0.545	0.917	0.508	0.446	0.226	0.182	0.202	0.852	0.342	0.060	**0.003**	**0.004**	**0.048**	0.076	0.695	0.749

**Table 6:** Circulating biomarkers in relation with the presence of signs of SVDs. Results are expressed as median (range).

		Circulating Biomarkers																					
		IL-6 (pg/mL)	IL-8 (pg/mL)	TNF-α (pg/mL)	IL-4 (pg/mL)	IL-10 (pg/mL)	CCL-2 (pg/mL)	CXCL-10 (pg/mL)	ICAM-1 (ng/mL)	VCAM-1 (ng/mL)	VEGF (pg/mL)	EMMPRIN (ng/mL)	MMP-2 (ng/mL)	MMP-7 (ng/mL)	MMP-8 (ng/mL)	MMP-9 (ng/mL)	MMP-12 (ng/mL)	TIMP-1 (ng/mL)	TIMP-2 (ng/mL)	TIMP-3 (ng/mL)	TIMP-4 (ng/mL)	VWF (%)	PAI-1 (ng/mL)
**SVDs**	**No sign** **(*n* = 28)**	0.84 (0.32–1.73)	6.02 (3.19–9.17)	2.03 (1.17–3.01)	6.5 (4.18–15.1)	3.07 (1.3–4.11)	288.8 (203.68–397.55)	14.9 (10.77–33.67)	316.1 (250.9–375.51)	1097.7 (888.16–1762.5)	51.74 (28.14–81.95)	5.4 (3.97–6.49)	**450.18** **(353.82–506.17)**	5.53 (3.62–7.37)	7.31 (1.98–11.9)	222.49 (150.85–395.63)	**249.35** **(49.5–836.28)**	**130.8** **(113.1–154.76)**	**108.38** **(88.35–123.6)**	**28.57** **(14.82–44.08)**	**2.58** **(2.19–3.55)**	162.1 (138.95–203.55)	9.01 (6.56–14.17)
	**1 sign** **(*n* = 39)**	12.81 (5–29.4)	8.33 (5.08–12.99)	2 (0.67–4)	12.81 (5–29.4)	3 (0.3–3.56)	16.33 (9.39–21.04)	16.33 (9.39–21.04)	341.23 (273.9–491.33)	1480 (1038.8–2044.3)	35.08 (36.8–106.75)	5.42 (4.03–6.46)	**500.59** **(421.8–622.53)**	5.57 (2.87–6.47)	6.68 (3.51–13.02)	315.35 (206.01–412.4)	**198.54** **(60.5–453.45)**	**159.12** **(126.86–216.77)**	**128.38** **(94.9–162.97)**	**31.21** **(21.22–47.68)**	**2.87** **(2.24–4.06)**	154 (125.32–204.25)	9.27 (6.92–16.35)
	**2 signs** **(*n* = 65)**	12.81 (5.3–36.03)	9.33 (5.08–13.81)	2.89 (1.06–4.92)	12.81 (5.3–36.03)	2.89 (0.32–3.46)	15.77 (9.75–22.47)	15.77 (9.75–22.47)	332.19 (256.86–569.97)	1691.7 (1007.7–2440.9)	72.79 (44.82–113.11)	5.8 (4.28–7.73)	**543.94** **(448.76–654.43)**	5.24 (3.27–6.71)	8.35 (3.43–16.54)	338.04 (185.63–527.69)	**450.1** **(132.64–612.28)**	**171.67** **(145–217.73)**	**134.07** **(110.3–236.56)**	**38.82** **(27.56–53.04)**	**3.34** **(2.36–4.93)**	170.1 (129.6–214.8)	9.35 (7.19–15.55)
	**≥3 signs** **(*n* = 34)**	12.8 (5.45–31.81)	8.73 (6.64–13.76)	2.04 (0.98–3.51)	12.81 (5.45–31.81)	3.05 (0.26–3.61)	14.51 (11.54–22.41)	14.51 (11.54–22.41)	325.94 (249.9–623.89)	1265.1 (1053.64–1763.13)	58.34 (38.17–110.6)	5.22 (3.45–7.48)	**574.16** **(476.54–690.86)**	5.35 (3.1–7.25)	6.84 (4.32–11.37)	297.74 (186.99–432.25)	**334.32** **(46.02–588.84)**	**149.1** **(120.37–203.73)**	**149.1** **(120.37–203.73)**	**45.07** **(29.36–65.14)**	**3.48** **(2.67–6.64)**	192.55 (136.53–220.58)	9.24 (6.8–14.24)
	***p***	0.093	0.074	0.478	0.657	0.749	0.307	0.816	0.794	0.066	0.273	0.836	**<0.001**	0.809	0.656	0.339	**0.041**	**<0.001**	**<0.001**	**0.019**	**0.039**	0.592	0.987

**Table 7:** Circulating multivariate logistic regression analysis adjusted for age, sex, CHA2DS2-VASc HAS-BLED and type of anticoagulant.

											Endpoint																											
	Non-Lacunar Infarct		Lacunar Infarct								Cerebral Microbleeds								WMH (Fazekas)											EPVS								
**Variable**	PAI-1		TIMP-1		TIMP-2	TIMP-3	vWF		IL-8		IL-8		**MMP-2**	TIMP-1	TIMP-2		TIMP-3		**MMP-2**		**TIMP-1**		**TIMP-2**		TIMP-3		**TIMP-4**		IL-6	MMP-12	**TIMP-1**	**TIMP-2**	**TIMP-3**	**TIMP-4**		IL-6		**IL-8**
**OR** (95% CI)	1.03 (0.99–1.07)		1.30 (0.92–1.84) #		1.40 (0.99–1.98) #	1.35 (0.95–1.92) #	1.44 (0.97–2.14) #		1.02 (0.98–1.06) #		1.27 (0.88–1.82)		**1.51** **(1.06–2.15)** **#**	1.14 (0.78–1.66)	1.38 (0.97–1.97) #		1.39 (0.96–2.02) #		**1.79** **(1.08–2.98)** **#**		**2.03** **(1.24–3.35)** **#**		**1.89** **(1.18–3.02)** **#**		1.45 (0.96– 2.2) #		**1.96** **(1.16–3.31)** **#**		1.79 (0.84–3.48) #	1.01 (0.68–1.48) #	**1.78** **(1.14–2.80)** **#**	**1.56** **(1.03–2.36)** **#**	**1.54** **(1.03–2.97)** **#**	**1.73** **(1.12–2.70)** **#**		1.63 (0.83–3.2) #		**1.61** **(1.07–2.42)**
***p***	0.180		0.144		0.056	0.093	0.068		0.292		0.201		**0.023**	0.506	0.079		0.081		**0.025**		**0.005**		**0.008**		0.081		**0.012**		0.133	0.979	**0.012**	**0.035**	**0.036**	**0.015**		0.156		**0.024**
	**Endpoint**																																					
	**SVD**																																					
**Variable**	**MMP-2**					MMP-12						**TIMP-1**						**TIMP-2**						TIMP-3						**TIMP-4**			IL-8					
	1 vs. 0	**2 vs. 0**		**≥3 vs. 0**		1 vs. 0		2 vs. 0		≥3 vs. 0		**1 vs. 0**		**2 vs. 0**		**≥3 vs. 0**		1 vs. 0		**2 vs. 0**		**≥3 vs. 0**		1 vs. 0		2 vs. 0		≥3 vs. 0		1 vs. 0	2 vs. 0	**≥3 vs. 0**	1 vs. 0		2 vs. 0		≥3 vs. 0	
**OR** (95% CI)	1.64 (0.78–3.5) #	**2.17** **(1.06–4.47)** **#**		**2.41** **(1.14–5.09)** **#**		0.82 (0.44–1.50) #		1.26 (0.73–2.17) #		0.98 (0.52–1.83) #		**2.60** **(1.11–6.06)** **#**		**4.43** **(1.94–10.1)** **#**		**4.09** **(1.74–9.59)** **#**		2.03 (0.87–4.75) #		**2.93** **(1.3–6.62)** **#**		**3.64** **(1.57–8.44)** **#**		1.04 (0.55–1.97) #		1.52 (0.86–2.72) #		1.81 (0.98–3.32) #		1.51 (0.63–3.59) #	2.02 (0.89–4.58) #	**2.71** **(1.17–6.28)** **#**	1.7 (0.89–3.23)		1.84 (0.97–3.49)		1.84 (0.93–3.66)	
***p***	0.194	**0.035**		**0.022**		0.518		0.415		0.979		**0.027**		**<0.001**		**0.001**		0.101		**0.010**		**0.003**		0.897		0.148		0.057		0.353	0.091	**0.020**	0.108		0.061		0.080	

^#^ for each SD increase.

## 4. Discussion

The main results of this study demonstrate that alterations in the degradation of the extracellular matrix, as documented by levels of MMP-2 and TIMPs (2; 3; 4), are associated, in a model adjusted for several confounding factors, with the occurrence of microbleeds and SVD in a population of AF patients on oral anticoagulants. 

The aim of the present study was to identify possible biomarkers able to discriminate thrombotic and bleeding risk in a cohort of AF patients under treatment with any type of oral anticoagulant. 

For this purpose, we chose different circulating biomarkers involved in different pathways such as extracellular matrix, inflammation and clotting activation and we correlated these parameters with cerebral lesions documented by MRI.

To date, the validity of models recommended for the stratification of the risk ratio between benefits and hemorrhage risk in anticoagulated AF patients is limited, and there is a great interest in detecting a combination of biological markers in order to improve the validity of scores actually used.

Recently, in order to explore the pathophysiological features of ischemic stroke in AF patients, the association between 268 plasma proteins and subsequent ischemic strokes in patients receiving oral anticoagulation have been investigated. The authors report an association between subsequent ischemic strokes and the proteins involved in fibrosis, cardiac dysfunction and vascular calcifications [23].

According to these observations, in our study, by analyzing the presence of cerebral lacunar infarcts detected by MRI, we demonstrate a positive and significant independent association between MMP-2, TIMP-1, TIMP-2, TIMP-3 and the presence of the lesions. Patients with AF have evidence of impaired matrix degradation, and extracellular matrix degradation proteinases have previously been associated with left atrial dilatation and can indirectly reflect a general burden of cardiac fibrosis and cardiomyopathy [24]. 

By analyzing the risk of bleeding in our patients’ cohort, and in particular the presence of cerebral microbleeds, we reported that patients with CMBs had higher circulating levels of TIMP-2 and TIMP-3 with respect to patients without CMBs, and this association also remained significant in a multivariate logistic analysis.

Clearly, MMPs and TIMPs appear to represent the pathway of remodeling/fibrosis.

MMPs belong to a family of endopeptidases involved in tissue remodeling, directly degrading extracellular matrix proteins. They are secreted by several cells, most prominently fibroblasts, and macrophages. In cardiovascular disease, MMPs have been associated with hypertension, arterial stiffness, and have often been used as markers for myocardial fibrosis. However, the role of MMPs in AF have been less studied.

In particular, MMP-2 degrades collagen IV, which is the major component of the basement membrane. It has been reported that MMP-2 plays an important role in angiogenesis and heart development. Elevated circulating levels of MMP-2, along with decreased circulating levels of TIMP-2, were significantly associated with AF risk according to the results of our study. The present results, to our knowledge, describe for the first time the prognostic role of MMP2 and TIMPs as a risk marker for microbleeds and SVD in patients with AF. The relation may be through potential direct mechanisms because these extracellular matrix degradation proteinases have previously been associated with left atrial dilatation, or potentially through less specific pathways, reflecting a general burden of cardiac fibrosis and cardiomyopathy.

Matrix metalloproteinases and TIMPs maintain a relative balance in normal tissues [25,26] and as this balance is disturbed, the degradation of extracellular matrix occurs. Growing evidence supports the phenomenon that MMPs and TIMPs maintain a balance in a normal state, and when broken, both MMPs and TIMPs are regulated via a positive feedback loop. For example, increasing MMPs breaks the balance between MMPs and TIMPs, and the body compensates by increasing TIMPs via a positive feedback mechanism.

In addition, existing studies suggest that inflammation may play an important role in the development and progression of SVD [27,28]. In our study, we found a significant association between high levels of IL-8 and the presence of EPVS, also demonstrated in a multivariate model. IL-8 is a chemoattractant cytokine and angiogenic factor produced by a variety of tissue and blood cells that is able to regulate multiple biological activities in endothelial cells, such as regulating endothelial cell growth, survival, migration and MMP-2 production as well [29].

These results suggest that we could identify, by venous blood samples obtained at the beginning of oral anticoagulation in AF patients, those at high risk of occurrence of microbleeds and SVD. Probably, compared with individual plasma biomarkers, clusters of interrelated biomarkers associated with cerebral SVD may better explain the underlying pathological processes.

Nevertheless, the present study has some limitations. Firstly, our patient population was carefully selected according to the inclusion criteria of the study, and the relatively small sample size posed some limitations to our study’s results. Further studies enrolling a larger number of patients are needed to confirm and extend our results. Secondly, the cross-sectional design of the study does not allow us to identify patients at more elevated risk of thrombotic and bleeding complications according to laboratory parameters. In our study, we take a picture of the biohumoral profile and the MRI burden of lesion of the enrolled patients. Therefore, we are not able to establish a cause–effect relationship between the presence of biomarkers and the development of SVD lesions. However, the association between metalloproteinases, TIMPs and SVD lesions, documented by MRI, may be ascribed to the degradation of extracellular matrix by MMPs and inflammation. In fact, different clinical studies demonstrated that metalloproteinases contributed to the degradation of the neurovascular matrix and the disruption of the tight junction proteins and the cerebrovascular basal lamina protein, further promoting brain injury [30,31].

However, at present there are no data about the role of metalloproteinases and their inhibitors in influencing the SVD lesions’ occurrence. Our study identified these molecules as possible markers of SVD lesions. The follow-up study results will enable us to further underpin the hypothesis of a significant role of metalloproteinases in inducing SVD lesions: the longitudinal analysis (at an 18-month follow-up) is in progress.

In conclusion, our results represent a potential innovative tool able to identify patients at risk of the potential bleeding or thrombotic complication of therapy which will worse prognosis. Furthermore, the clinical relevance of these results is due to the fact that we have no efficient methods to identify bleeding risk in these patients. Indeed, if CHA2DS2-VASc is a clinical score which identifies with a good capacity to discriminate AF patients at low risk of stroke, HAS-BLED or any of the other available scores do not have the same power in the identification of patients who will suffer from bleeding complications.

## Figures and Tables

**Table 1:** Demographic and clinical characteristics of the baseline Strat-AF study cohort (*n* = 170). Results are expressed as median ± DS and as percentages.

Demographic and Clinical Characteristics	Total Cohort *n* = 170
Age (years)	77.7 ± 6.8
Gender (females)	59 (34.7%)
Schooling (years)	9.1 ± 4.3
Stroke	38 (22.4%)
Coronary artery disease	18 (10.6%)
Heart failure	25 (14.7%)
Peripheral arterial pathology	14 (8%)
Hypertension	140 (82.4%)
Diabetes	22 (12.9%)
Dyslipidemia	87 (51.2%)
Physical activity (lack of)	110 (64.7%)
Smoke	105 (61.8%)
Alcohol consumption	91 (53.5%)
BMI (kg/m^2^)	26.3 ± 3.9
CHA₂DS₂-VASc Score	3.69 ± 1.49
HAS-BLED	1.85 ± 0.89

## Data Availability

The data presented in this study are available on request from the corresponding author. The data are not publicly available due to privacy or ethical restrictions.
